# Supplementary material for: 3D printing of thermosets with diverse rheological and functional applicabilities
Source: Nat Commun. 2023 Jan 16;14:245. doi: 10.1038/s41467-023-35929-y (PMC9842742; doi:10.1038/s41467-023-35929-y)
Supplement: Supplementary file 1 — Supplementary Information [file 41467_2023_35929_MOESM1_ESM.pdf]

Supplementary Information

3D printing of thermosets with diverse rheological and functional applicabilities

Yuxuan Sun[1], Liu Wang[2]*, Yangyang Ni[1], Huajian Zhang[1], Xiang Cui[3], Jiahao Li[2], Yinbo Zhu[2], Ji Liu[4], Shiwu Zhang[1], Yong Chen[5]*, Mujun Li[1]*

1. Department of Precision Machinery and Precision Instrumentation, University of Science and Technology of China, Hefei 230026, Anhui, China

2. CAS Key Laboratory of Mechanical Behavior and Design of Materials, Department of Modern Mechanics, University of Science and Technology of China, Hefei 230026, Anhui, China

3. School of Computer Science and Technology, University of Science and Technology of China, Hefei 230026, Anhui, China

4. Department of Mechanical and Energy Engineering, Southern University of Science and Technology of China, Shenzhen 518055, China

5. Epstein Department of Industrial and Systems Engineering, Viterbi School of Engineering, University of Southern California, Los Angeles, CA 90089, US

*Corresponding authors.

E-mail: wangliu05@ustc.edu.cn (L.W); yongchen@usc.edu (Y.C); lmn@ustc.edu.cn (M.L).

Supplementary Note 1

Modeling of the crosslinking and viscosity of thermosetting ink

The crosslinking kinetics of thermosetting ink can be described by the autocatalytic model shown in Supplementary Eq. 1:

$$\frac{\mathrm{d}p}{\mathrm{d}t} = k(T)p^m(1-p)^n \qquad \text{Supplementary Eq. 1}$$

where p is the crosslink extent, dp/dt is the crosslinking rate, m and n are partial reaction orders, the term pm represents the availability of catalyst, while $(1-p)^n$ models the amount of reagent, or the available functional group for crosslinking. The term $k(T)$ described by the Arrhenius function is the same as $k(T)$ in Eq. 1. The crosslinking of thermosets is an exothermic reaction:

$$p = \frac{Q}{Q_T} = (\int_0^{\tau} \dot{Q}\mathrm{d}t)/(\int_0^{\tau_f} \dot{Q}\mathrm{d}t) \qquad \text{Supplementary Eq. 2}$$

where Q is the heat released up to time τ, Q_T is the total heat released and $\dot{Q}$ is the rate of heat release. Q and $\dot{Q}$ are measured by DSC and the change of p under different temperature conditions can directly calculated. Then, the autocatalytic model parameters of Sylgard 184 were calculated according to ASTM E2070-08. Upon poisoning, the silicone yield p = 0 crosslinking extend, therefore Eq. 1 is changed into:

$$\ln(\eta) = \ln(\eta_\infty) + \frac{E_{a,\eta}}{RT} \qquad \text{Supplementary Eq. 3}$$

By measuring the viscosity change with the temperature of poisoned Sylgard 184, the parameters in Supplementary Eq. 3 could be fitted. The calculated parameters for crosslinking and viscosity change are listed in Supplementary Table 3 and Table 4.

Mechanical and magnetic properties of magnetic soft composite

The magnetization strength of magnetic composite (M) is linearly proportional to the volume fraction of NdFeB particles (denoted as φ), which is expressed as:

$$M = M_p \varphi \quad \text{Supplementary Eq. 4}$$

where $M_p = 640$ kA/m is the magnetization strength of NdFeB. The shear modulus G can be calculated by the Mooney model:

$$G = G_0 \exp\left(\frac{2.5\varphi}{1-1.35\varphi}\right) \quad \text{Supplementary Eq. 5}$$

where $G_0 = 2.8MPa$ is the shear modulus of pure silicone.

Supplementary Table 1. Comparison between ISDH and existing manufacturing approaches of thermosets

Approach	Material Requirements	Manufacturing cycle time*	Geometric complexity	Facilities	Cost
Casting[3,8-10]	Low viscosity resin	Days	Low	Oven, mold	Intermediate
Compression molding[1]	Intermediate semi-cured composite	Weeks	Low	Hydraulic press, autoclave, mold	Expensive
Reaction injection molding[1,3]	Low viscosity resin	Weeks	Low	Injection molding machine, mold	Expensive
**ISDH 3D printing (This work)**	**Diverse rheological properties**	**Hours**	**High**	**Modified from commercial FDM 3D printers**	**Low cost**

*Time cost includes the preparation (e.g., model/mold design), fabrication, and post-processing (e.g., demolding, dissolving bath-support)

Supplementary Table 2. Comparison of different approaches for 3D printing of thermosets

Type	Mechanism	Minimum nozzle /resolution (µm)	Printable height	Material applicability	Tailored thermosets property	Multi-functionality	Hybrid printing
Vat polymerization	Direct sound printing by sonochemical reaction[18]	None/280	High	Limited inks with specific porosity	No	No	No
	DLP or SLA by light-curing[14-17]	None/20	High	Light-curable resin	No	Yes	Yes
Direct ink writing	Rheology modification by adding particles [23-29]	50/50	Low	Yield-stress	Yes	Yes	Limited
	Self-assembled yield-stress polymers[23,24]	150/150	Low	Yield-stress	Yes	No	Limited
	Embedded printing in bath-support [33-36]	100/100	High	Newtonian, shearing-thinning, yield-stress	No	Limited	No
	Frontal polymerization by chemical reaction[38-40]	250/250	High	Limited to dicyclopentadiene	Yes	No	Limited
	In-situ light curing[41,42]	100/100	High	Light-curable resin	No	Yes	Yes
	***In situ* gelation by ISDH (This work)**	**25/50**	**High**	**Newtonian, shearing-thinning, yield-stress**	**No**	**Yes**	**Yes**

Supplementary Table 3. Crosslinking and viscosity parameters of Sylgard 184

R(J·mol^{-1}K^{-1})	k_0(s^{-1})	$E_{a,k}$(J/mol)	m	n	η_∞(Pa·s)	$E_{a,k}$(J)
8.314	5.8569×10^7	6.2696×10^4	1.1536	1.1589	0.0118	-75582.6

Supplementary Table 4. Crosslinking and viscosity parameters of Sylgard 184

h_g (W·m^{-2}k^{-1})	h_c (W·m^{-2}k^{-1})	ρ_{PDMS} (kg·m^{-3})	ρ_{air} (kg·m^{-3})	ε_{heater}	ε_{PDMS}	k_{PDMS} (W·m^{-1}k^{-1})	k_{air} (W·m^{-1}k^{-1})
20	1000	1030	a)	0.92	1	0.16	b)

a) The density of air is $0.02897P/RT$;

b) The coefficient of thermal conductivity of air is:

$-0.0022758+1.1538002\times10^{-4}T-7.9025286\times10^{-8}T^2+4.1170251\times10^{-11}T^3-7.4386433\times10^{-15}T^4$

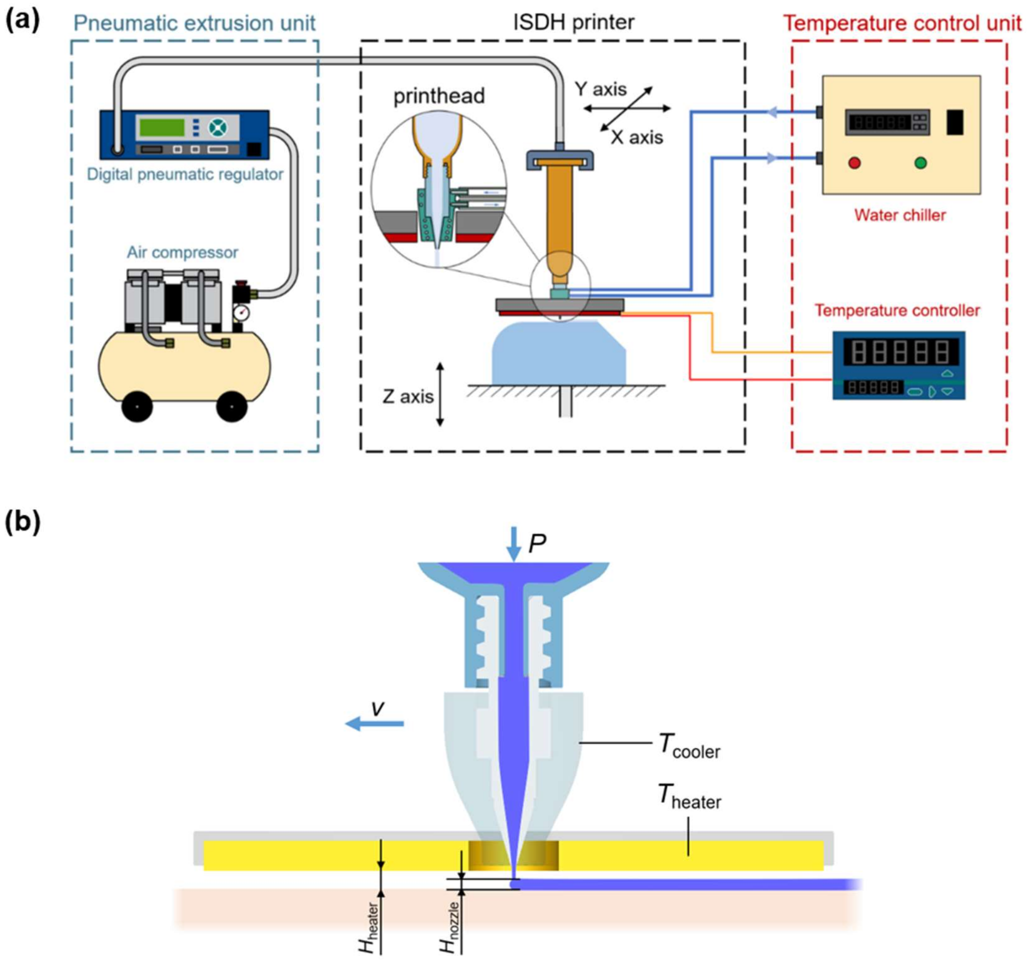

Supplementary Fig. 1. The overall setup of ISDH 3D printing based on a commercial 3D printer. **a** The system includes a ISDH printer (modified from a commercial FDM printer with a home-build printhead), a pneumatic extrusion unit (including an air compressor and a digital pneumatic regulator) to provide air pressure, and temperature control unit, which consists of a water chiller to provide circulated cooling water and a temperature controller for PID control the temperature of the Joule heater. **b** Control parameters for ISDH printing. Key parameters including the height of the nozzle and heater with respect to the cured ink base (H_{nozzle} = 0.3 mm and H_{heater} = 1mm), the temperature of the heater and cooler (T_{heater} and T_{cooler}), air pressure applied for ink extrusion (P) and moving speed of the nozzle (v=30 mm/s).

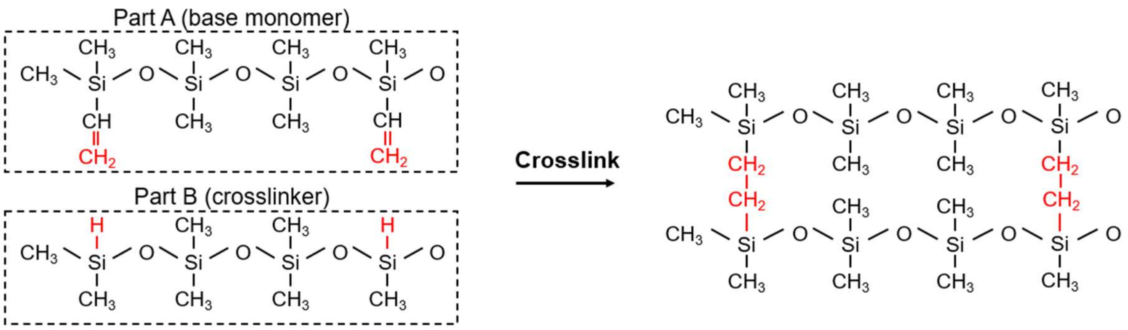

Supplementary Fig. 2. Chemical composition and crosslinking reaction of Sylgard 184.

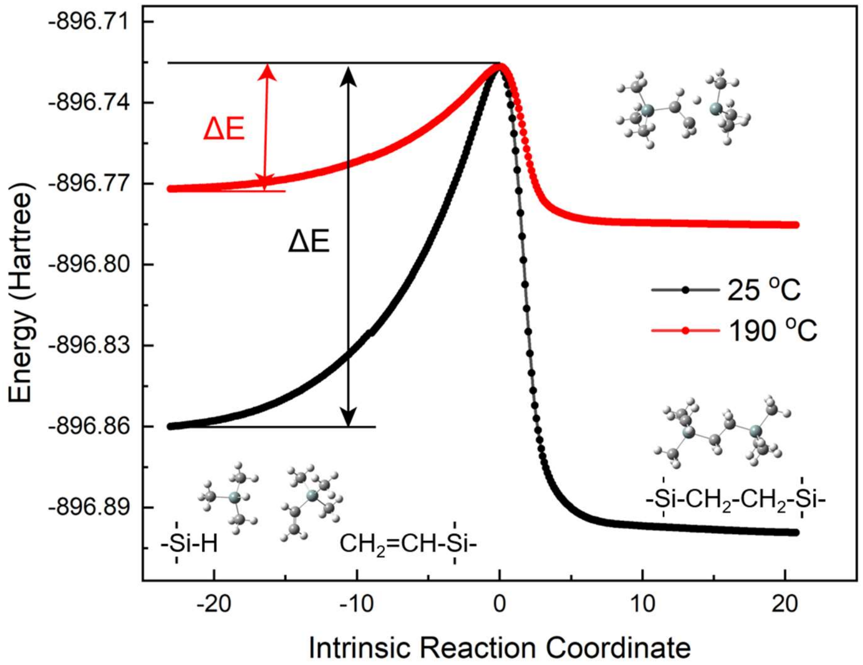

Supplementary Fig. 3. DFT simulation of crosslinking of Sylgard 184 in which (-Si-CH=CH_2 + H-Si- → -Si-CH_2-CH_2-Si-). The energy barrier ($\triangle E$) at 190°C (red curve) is significantly reduced compared with that at 25°C (black curve).

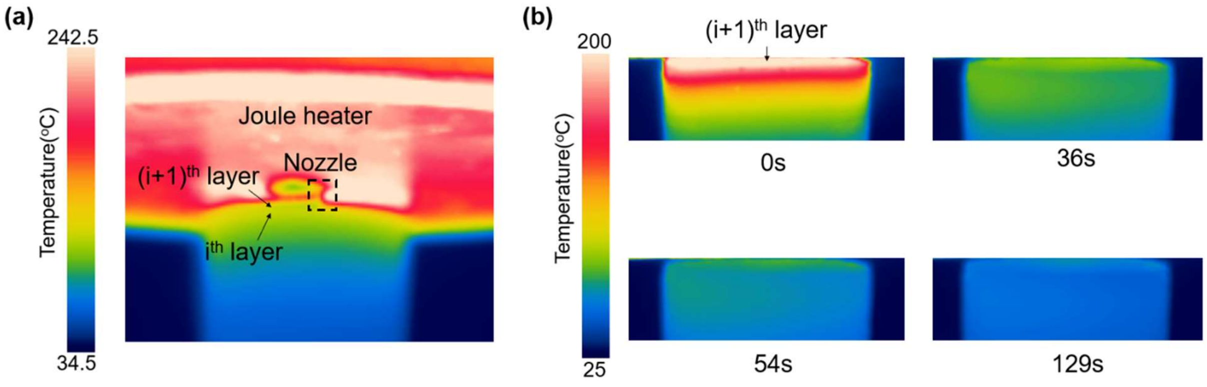

Supplementary Fig. 4. Infrared camera image of the temperature distribution during ISDH printing process. **a** Temperature distribution during ISDH printing. **b** Temperature of (i+1)th layer after heater removed for 0s, 36s, 54s and 129s.

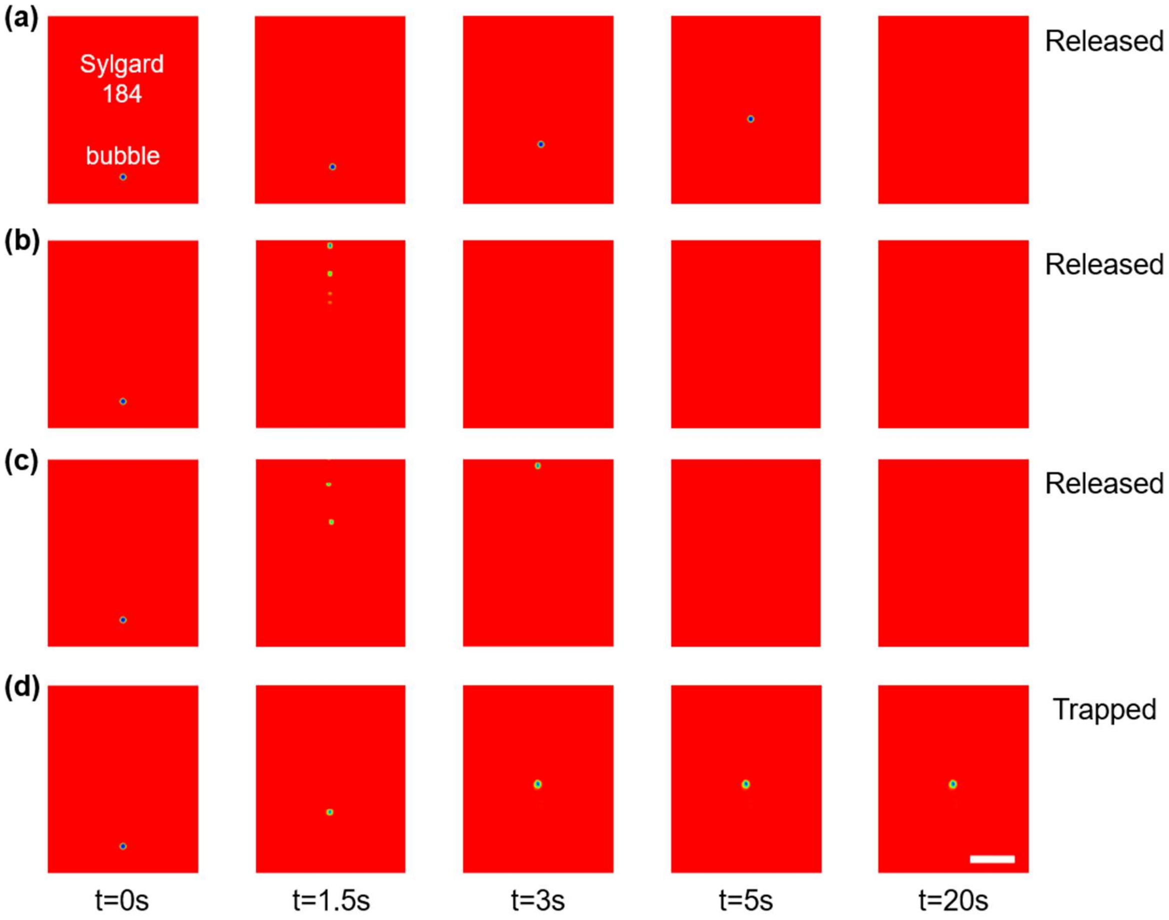

Supplementary Fig. 5. CFD simulation of degassing under different temperatures using a 300-um-diameter nozzle. **a** Image sequences of an escaping bubble at 50 °C. **b** Image sequences of an escaping bubble at 100°C. **c** Image sequences of an escaping bubble at 190°C. **d** Image sequences of a trapped bubble at 220 °C. (scale bar=50 μm)

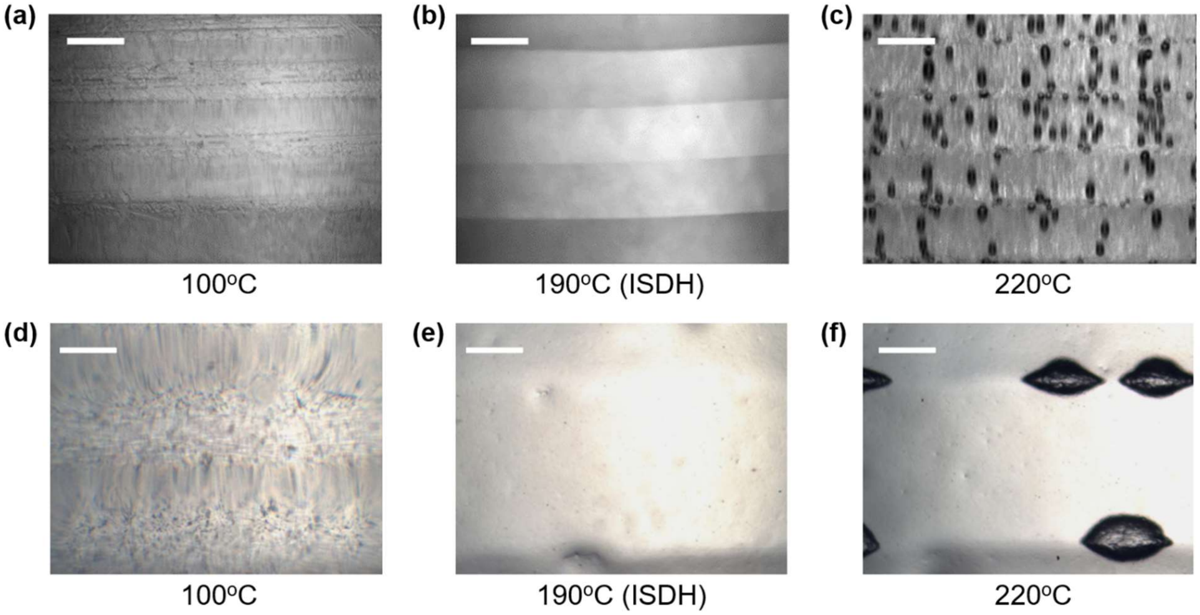

Supplementary Fig. 6. Optical microscope image of printed Sylgard 184 under different conditions. **a-c** Nozzle diameter 300 μm at **a** 100 °C; b 190 °C; **c** 220 °C. **d-f** Nozzle diameter 1mm at **d** 100 °C; **e** 190 °C; **f** 220 °C. (Scale bar=300 μm)

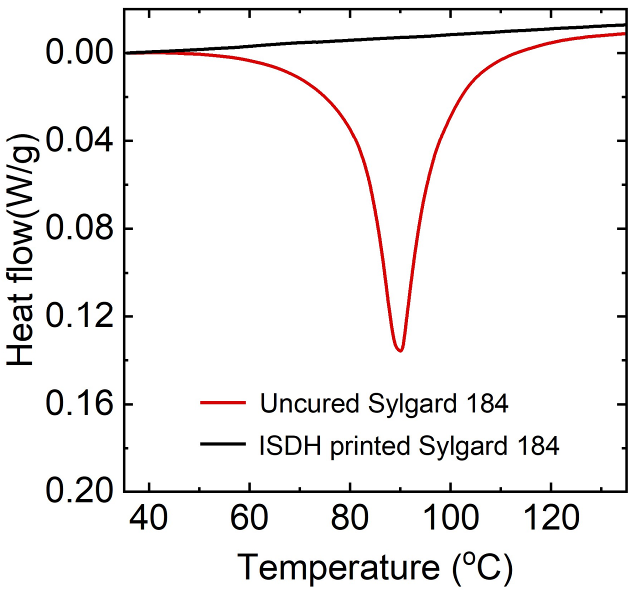

Supplementary Fig. 7. DSC curve of uncured and ISDH printed Sylgard 184. The degree of cure is calculated as 99.4%.

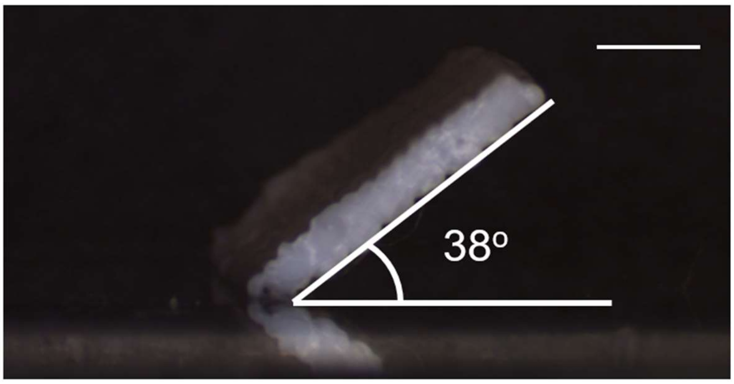

Supplementary Fig. 8. Cross-sectional images of a Sylgard 184 wall with a 38° incline angle (scale bar=2 mm).

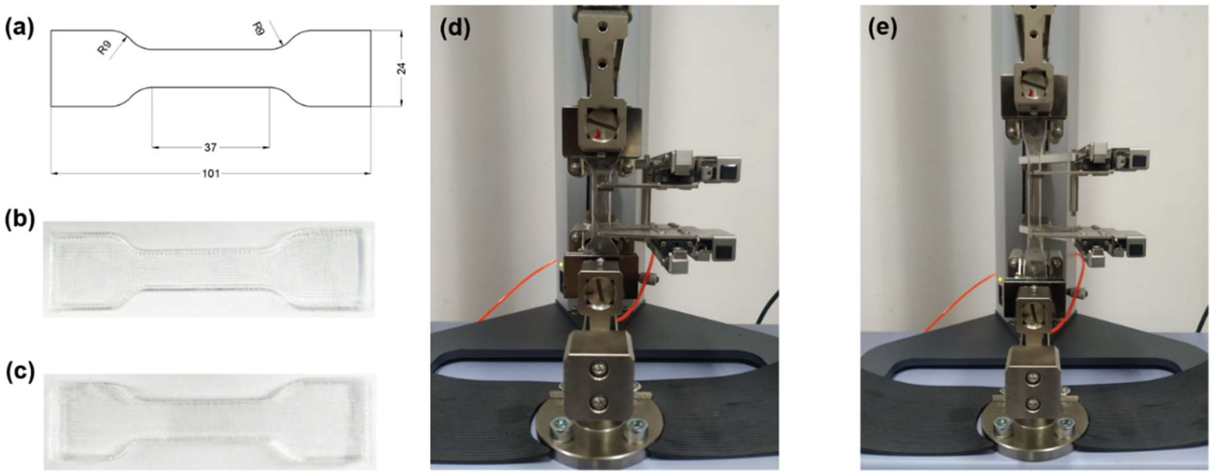

Supplementary Fig. 9. ISDH printed tensile specimen and tensile test. **a** Design of tensile specimen. **b** ISDH printed specimen with printing direction parallel to tensile direction. **c** ISDH printed specimen with printing direction perpendicular to tensile direction. **d** and **e** Tensile experiment of ISDH printed Sylgard 184 specimen.

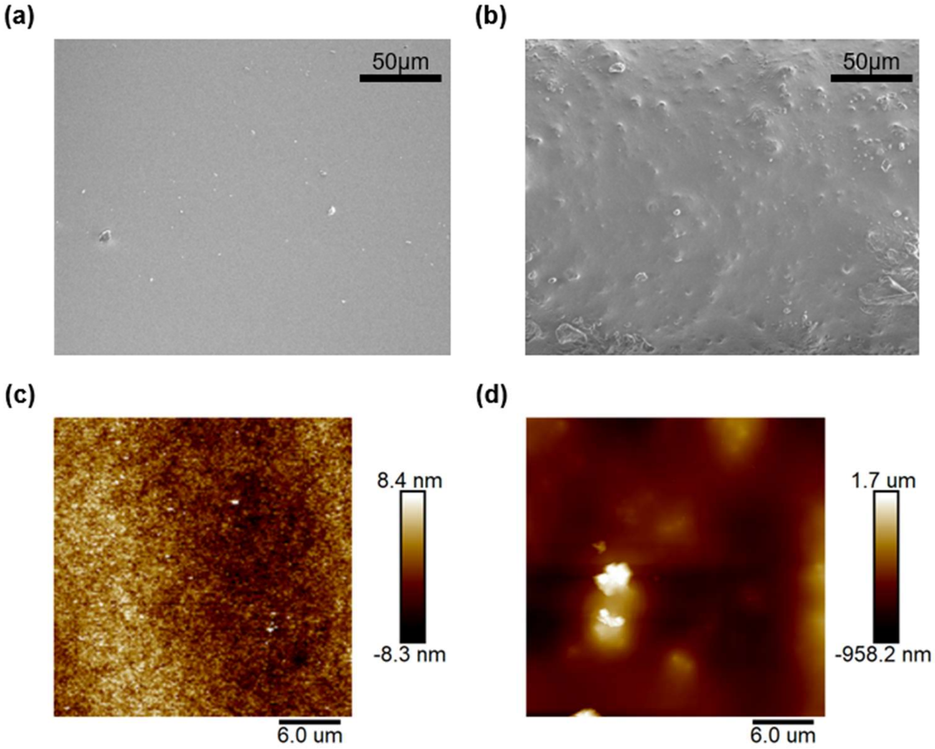

Supplementary Fig. 10. Surface comparison of ISDH printed pure silicone and DIW printed fumed silica modified silicone (5 wt% fumed silica). **a** and **c** SEM and AFM images of ISDH printed Sylgard 184. **b** and **d** SEM and AFM images of DIW printed Sylgard with 5 wt% fumed silica.

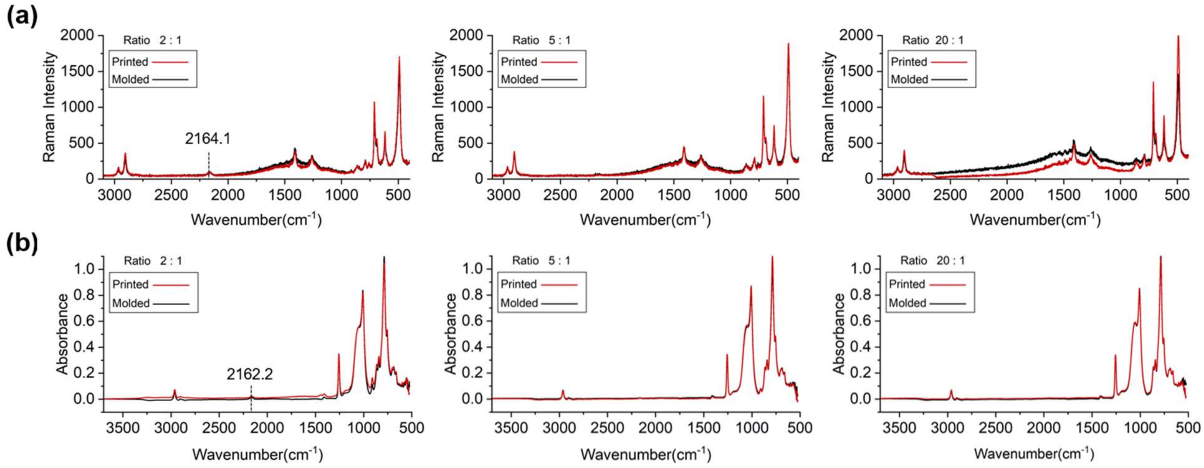

Supplementary Fig. 11. Comparison between ISDH-printed and molded samples using Sylgard 184 with different mixing ratios of Part A and B. **a** Raman spectroscopy; **b** infrared spectroscopy.

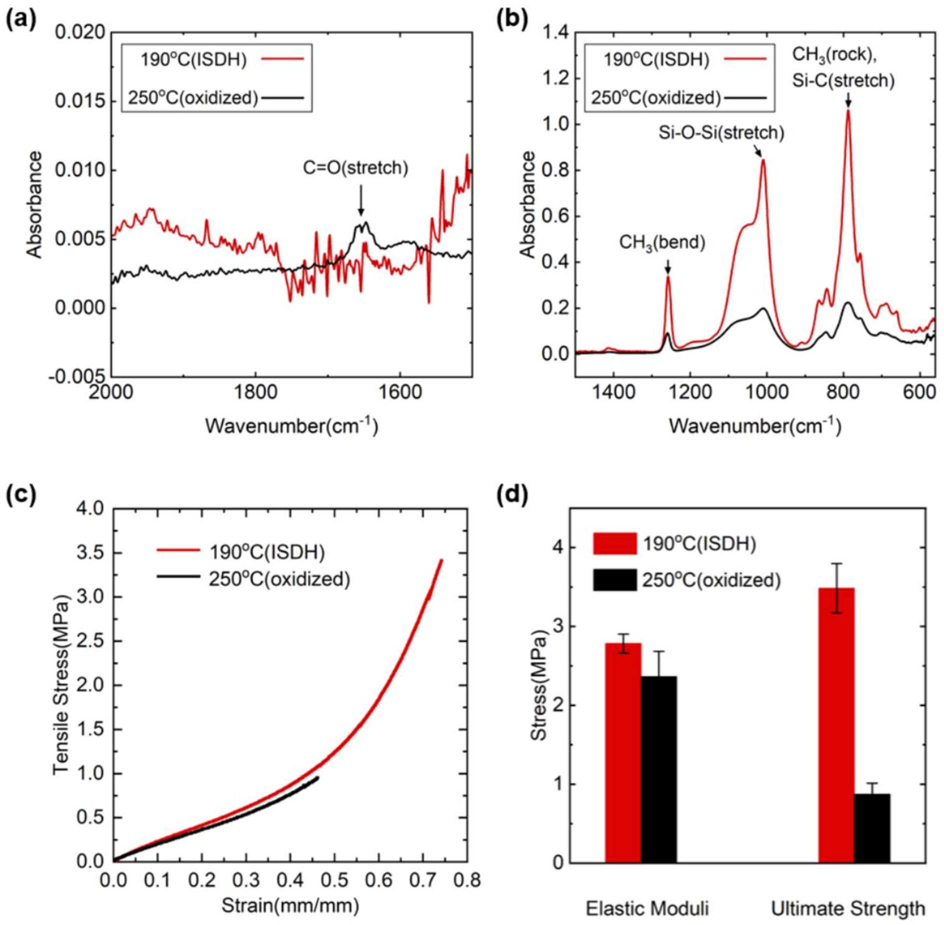

Supplementary Fig. 12. Comparison of ISDH printed Sylgard 184 and oxidized samples at 250°C. **a** and **b** Infrared spectroscopy of ISDH printed and oxidized samples. Result shows existence of C=O band and lower CH_3, Si-O-Si and Si-C absorbance in the oxidized sample. **c** and **d** mechanical properties of ISDH printed and oxidized samples, result shows lower elastic moduli and ultimate strength of oxidized samples.

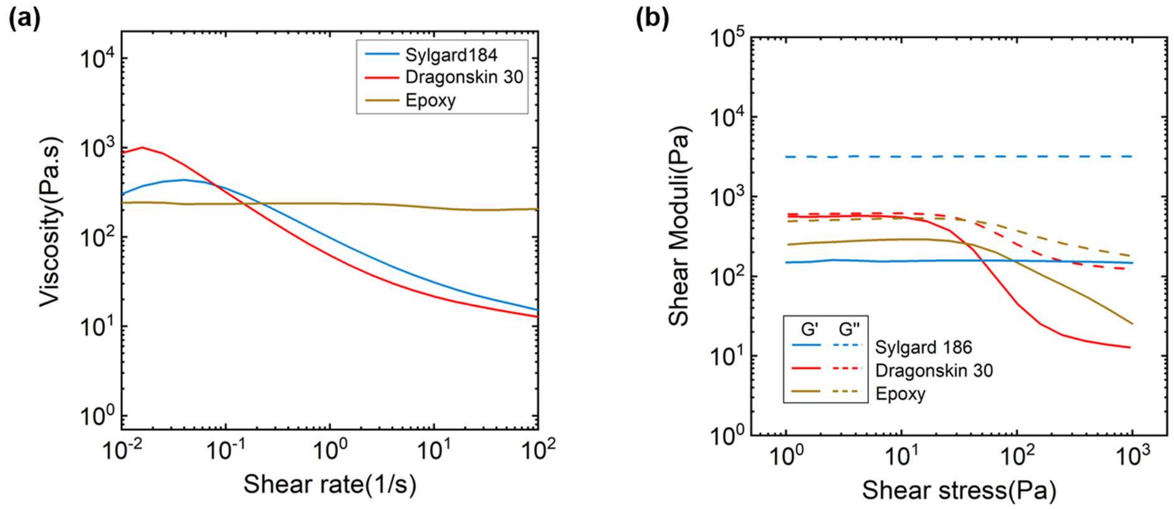

Supplementary Fig. 13. Rheological properties of Sylgard186, Dragonskin 30, and epoxy. **a** Viscosity under different shear rates. **b** Storage and loss moduli under different shear stresses.

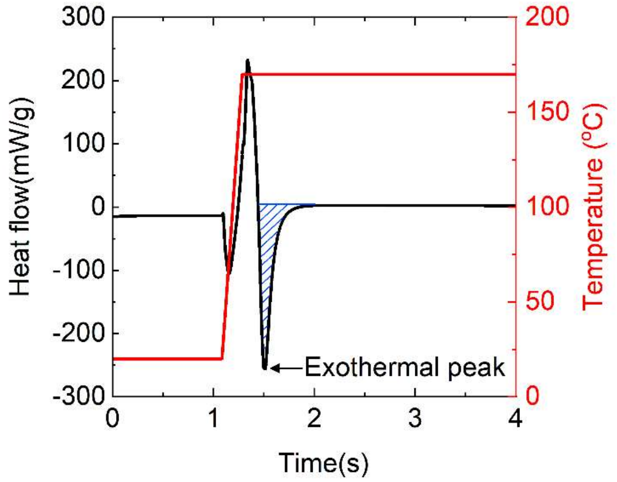

Supplementary Fig. 14. DSC curve of epoxy under 170°C. The blue area marks exothermal of crosslinking reaction. Results show the crosslinking of epoxy finishes within 1s after heating from room temperature to 170°C.

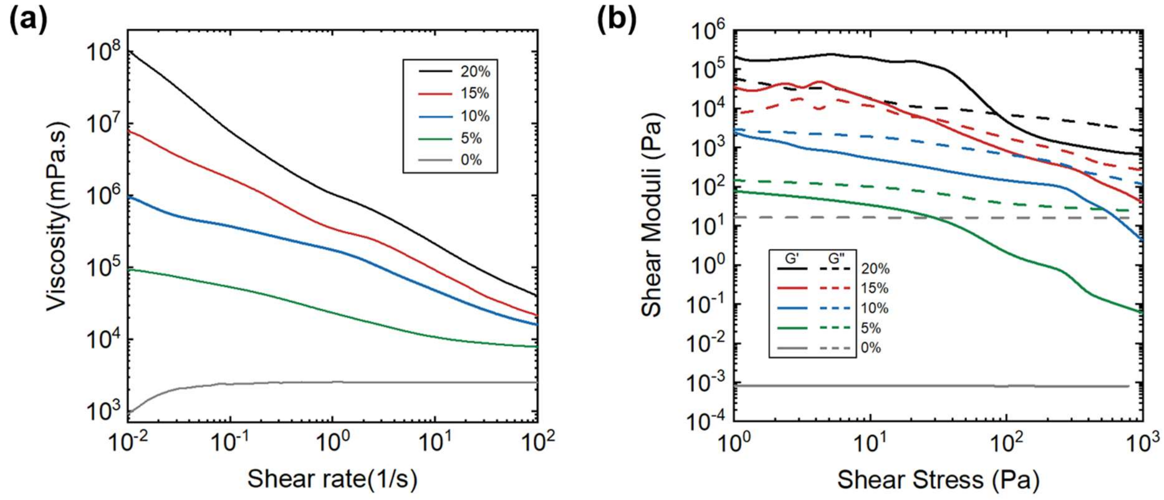

Supplementary Fig. 15. Rheological properties of magnetic composites with Sylgard 184 prepolymer and different contents of NdFeB particles. **a** Viscosity under different shear rates. **b** Storage and loss moduli under different shear stresses.

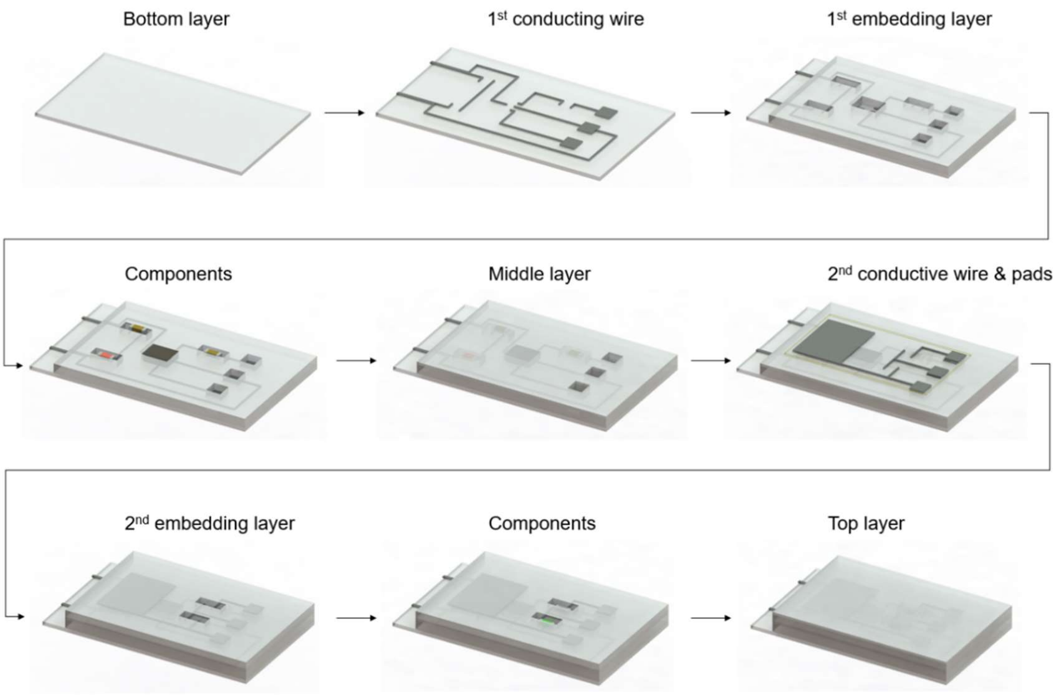

Supplementary Fig. 16. Hybrid printing process of the multilayer soft touch sensor.
