# Supplementary material for: 3D printing of thermosets with diverse rheological and functional applicabilities
Source: Nat Commun. 2023 Jan 16;14:245. doi: 10.1038/s41467-023-35929-y (PMC9842742; doi:10.1038/s41467-023-35929-y)
Supplement: Supplementary file 3 — Description of Additional Supplementary Files [file 41467_2023_35929_MOESM3_ESM.pdf]

Description of supplementary files

Supplementary Movie 1. Demonstration of printing a tube using low-viscosity Sylgard 184
Supplementary Movie 2. Demonstration of printing the magnetic stent
Supplementary Movie 3. Actuation of ISDH-printed functional thermosets using magnetic composites
Supplementary Movie 4. Demonstration of soft touch sensor
